# Supplementary material for: Altered microRNA Transcriptome in Cultured Human Liver Cells upon Infection with Ebola Virus
Source: Int J Mol Sci. 2021 Apr 6;22(7):3792. doi: 10.3390/ijms22073792 (PMC8038836; doi:10.3390/ijms22073792)
Supplement: Supplementary file 1 [file ijms-22-03792-s001.zip › Supplementary_File/C_ GO_Analysis_Results/16-30nt_go_Reston-96h-Huh7_vs_Control-96h-Huh7_up.mature_mirna_targets/GeneOntology_EnrichmentScore.pdf]

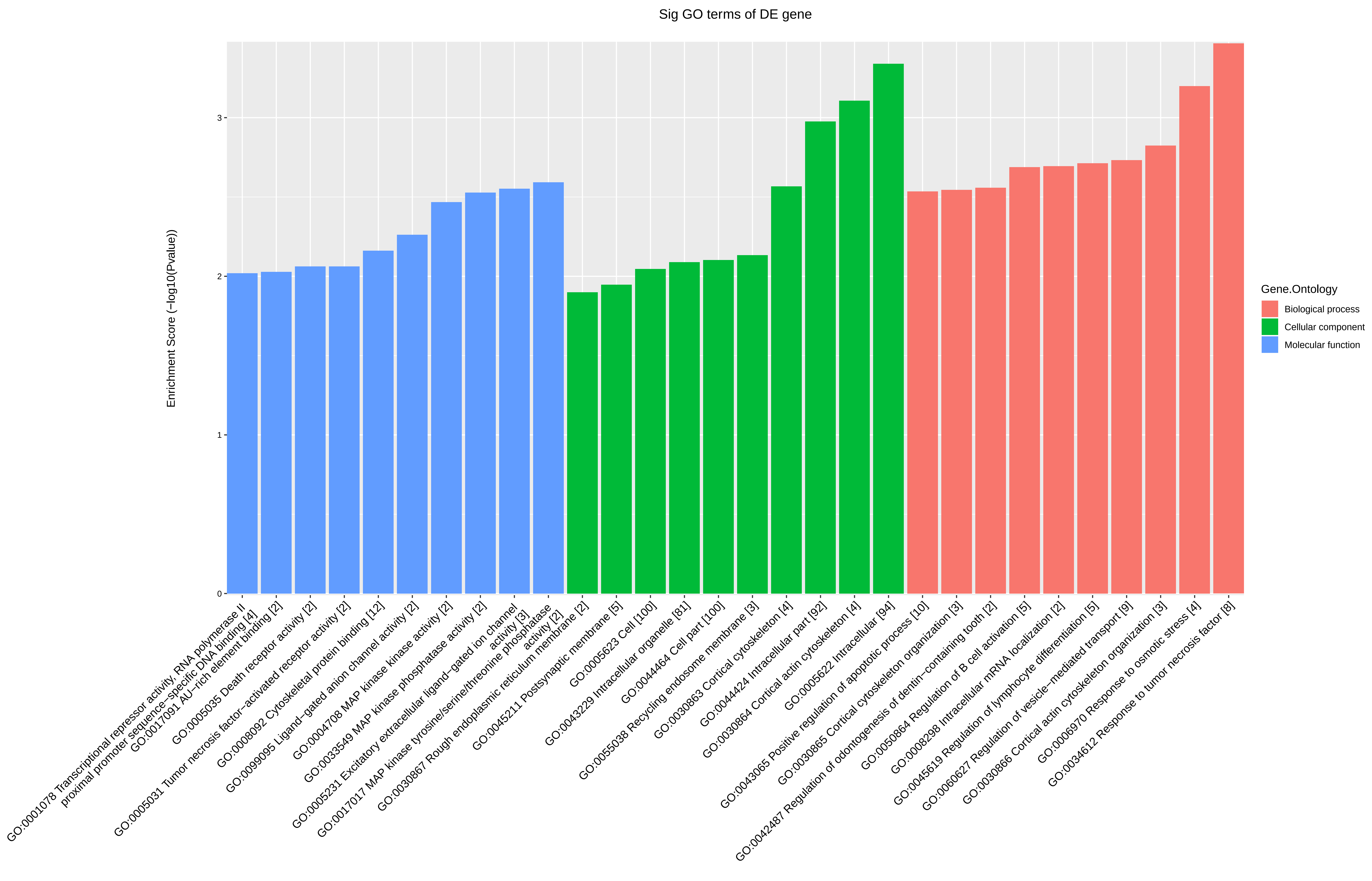

Sig GO terms of DE gene
Enrichment Score (−log10(Pvalue))
0
1
2
3
Gene.Ontology
Biological process
Cellular component
Molecular function
GO:0001078 Transcriptional repressor activity, RNA polymerase II proximal promoter sequence−specific DNA binding [4]
GO:0017091 AU−rich element binding [2]
GO:0005035 Death receptor activity [2]
GO:0005031 Tumor necrosis factor−activated receptor activity [2]
GO:0008092 Cytoskeletal protein binding [12]
GO:0099095 Ligand−gated anion channel activity [2]
GO:0004708 MAP kinase kinase activity [2]
GO:0033549 MAP kinase phosphatase activity [2]
GO:0005231 Excitatory extracellular ligand−gated ion channel activity [3]
GO:0017017 MAP kinase tyrosine/serine/threonine phosphatase activity [2]
GO:0030867 Rough endoplasmic reticulum membrane [2]
GO:0045211 Postsynaptic membrane [5]
GO:0005623 Cell [100]
GO:0043229 Intracellular organelle [81]
GO:0044464 Cell part [100]
GO:0055038 Recycling endosome membrane [3]
GO:0030863 Cortical cytoskeleton [4]
GO:0044424 Intracellular part [92]
GO:0030864 Cortical actin cytoskeleton [4]
GO:0005622 Intracellular [94]
GO:0043065 Positive regulation of apoptotic process [10]
GO:0030865 Cortical cytoskeleton organization [3]
GO:0042487 Regulation of odontogenesis of dentin−containing tooth [2]
GO:0050864 Regulation of B cell activation [5]
GO:0008298 Intracellular mRNA localization [2]
GO:0045619 Regulation of lymphocyte differentiation [5]
GO:0060627 Regulation of vesicle−mediated transport [9]
GO:0030866 Cortical actin cytoskeleton organization [3]
GO:0006970 Response to osmotic stress [4]
GO:0034612 Response to tumor necrosis factor [8]
